# The Impact of COVID-19 Confinement on Tinnitus and Hearing Loss in Older Adults: Data From the LOST in Lombardia Study

**DOI:** 10.3389/fneur.2022.838291

**Published:** 2022-03-07

**Authors:** Carlotta Micaela Jarach, Alessandra Lugo, Chiara Stival, Cristina Bosetti, Andrea Amerio, Luca Cavalieri d'Oro, Licia Iacoviello, Anna Odone, David Stuckler, Alberto Zucchi, Piet van den Brandt, Werner Garavello, Christopher R. Cederroth, Winfried Schlee, Silvano Gallus, The LOST in Lombardia Study Investigators, Silvano Gallus

**Affiliations:** ^1^Department of Environmental Health Sciences, Istituto di Ricerche Farmacologiche Mario Negri IRCCS, Milan, Italy; ^2^Department of Oncology, Istituto di Ricerche Farmacologiche Mario Negri IRCCS, Milan, Italy; ^3^Department of Neuroscience, Rehabilitation, Ophthalmology, Genetics, Maternal and Child Health (DINOGMI), Section of Psychiatry, University of Genoa, Genoa, Italy; ^4^IRCCS Ospedale Policlinico San Martino, Genoa, Italy; ^5^ATS della Brianza, Monza, Italy; ^6^Research Center in Epidemiology and Preventive Medicine (EPIMED), Department of Medicine and Surgery, University of Insubria, Varese, Italy; ^7^Department of Epidemiology and Prevention, IRCCS Neuromed, Pozzilli, Italy; ^8^School of Medicine, University Vita-Salute San Raffaele, Milan, Italy; ^9^Department of Public Health, Experimental and Forensic Medicine, University of Pavia, Pavia, Italy; ^10^Department of Social Sciences and Politics, Bocconi University, Milan, Italy; ^11^ATS di Bergamo, Bergamo, Italy; ^12^Department of Epidemiology, GROW-School for Oncology and Developmental Biology, Maastricht University Medical Centre, Maastricht, Netherlands; ^13^Department of Epidemiology, CAPHRI-School for Public Health and Primary Care, Maastricht University Medical Centre, Maastricht, Netherlands; ^14^Department of Otorhinolaryngology, School of Medicine and Surgery, University of Milan-Bicocca, Milan, Italy; ^15^Laboratory of Experimental Audiology, Department of Physiology and Pharmacology, Karolinska Institutet, Stockholm, Sweden; ^16^Nottingham Biomedical Research Centre, National Institute for Health Research (NIHR), Nottingham University Hospitals NHS Trust, Nottingham, United Kingdom; ^17^Division of Clinical Neuroscience, Hearing Sciences, School of Medicine, University of Nottingham, Nottingham, United Kingdom; ^18^Department of Psychiatry and Psychotherapy, University Regensburg, Regensburg, Germany

**Keywords:** tinnitus, hearing loss, older adults, COVID-19, cross-sectional study

## Abstract

**Background:**

Although a direct relationship between tinnitus or hearing difficulties and COVID-19 has been suggested, current literature provides inconsistent results, and no research has been undertaken in older adults.

**Methods:**

In November 2020, we conducted the LOST in Lombardia survey, a telephone-based cross-sectional study on a sample of 4,400 individuals representative of the general population aged ≥65 years from Lombardy region, Northern Italy. Individuals with diagnosed tinnitus and/or hearing loss were asked whether their conditions had improved or deteriorated in 2020 compared to 2019.

**Results:**

Overall, 8.1% of older adults reported a diagnosis of tinnitus and 10.5% of hearing loss. In 2020 compared to 2019, among individuals with tinnitus, those with increasing severity (5.0%) were similar to those decreasing it (5.3%). Among individuals with hearing loss, more people reported an increase (13.6%) than a decrease (3.2%) in their disease severity. No individual with a diagnosis in 2020 of tinnitus (*n* = 6) or hearing loss (*n* = 13) had COVID-19. The incidence of tinnitus was lower in 2020 (rate: 14.8 per 10,000 person-years) than in previous years (rate in 1990–2019: 36.0 per 10,000 person-years; *p* = 0.026). There was no change in the incidence of hearing loss (*p* = 0.134).

**Conclusions:**

In this large representative sample of older adults, on average neither COVID-19 confinement nor SARS-CoV-2 infection appeared to increase the severity or incidence of tinnitus. The increased severity of hearing difficulties may totally or partially be explained by physiologic deterioration of the condition, or by a misperception due to the use of face-masks.

## Introduction

When the first cases of SARS-CoV-2 were diagnosed in Lombardy in February 2020 (1), Italy became the first country in Europe to be hit by COVID-19. Lombardy remained the Italian area most struck by the pandemic, particularly in its early stages, reporting the largest number of infections and the highest hospital congestion (2, 3). In Italy and throughout the world, confinement has influenced not only the healthcare system and the economy, but also the lives and mental health of millions of individuals, raising their levels of anxiety and depressive symptoms (4, 5). People with tinnitus are one at-risk category for these mental health complications (5, 6). More than a disease, tinnitus is a symptom of underlying problems that describes the perception of noises in the brain or ears when there are no corresponding external acoustic stimuli (7, 8).

Given the direct relationship with mental health outcomes (5, 6), aggravation of tinnitus or a rise in its incidence has been hypothesized in tinnitus sufferers after the COVID-19 crisis (5). Since females have been shown to be more susceptible than males to mental health consequences during the COVID-19 pandemic (4), an increase in tinnitus severity can be expected particularly in women. Thus, a few cohorts of tinnitus patients have shown an increase in tinnitus severity, assessed through validated questionnaires, possibly promoted by frustration or anxiety (9, 10).

Beukes and colleagues conducted a systematic review to understand the influence of the COVID-19 pandemic or SARS-CoV-2 infection on tinnitus (11). Although this systematic review included 33 studies, many of the research questions remained unanswered. In fact, no study evaluated the impact of the pandemic on the incidence of tinnitus, or on the severity of tinnitus, while changes in tinnitus severity were inferred from the findings of only a few investigations (5, 9, 10). Results were inconclusive on the role of SARS-CoV-2 infection on the occurrence, duration, or severity of tinnitus (11).

Tinnitus is strongly associated with hearing loss (12), so an increase in hearing loss diagnoses could also be speculated. The widespread use of facial masks to prevent infection might have made hearing difficulties more severe: lip reading was not possible, and transmission of sound was reduced by the mask as a physical barrier, thus patients might have experienced deterioration in their hearing difficulties (11, 13). Moreover, various data suggest that hearing loss might be an audiological consequence and clinical manifestation of SARS-CoV-2 infection (14–17). Accordingly, a higher incidence of these conditions in 2020 than in previous years, or a higher prevalence in COVID-19 patients, might be due to the ototoxicity of some medications, and could be expected (16). However, the issue is still debated (18).

The purpose of this study is to assess the effects of confinement due to the COVID-19 pandemic on tinnitus and hearing loss in older adults in the Lombardy area.

## Methods

We used data from a telephone-based cross-sectional survey performed by Doxa, the Italian division of the Worldwide Independent Network/Gallup International Association, and coordinated by the Mario Negri Institute and other Italian universities and research institutions (19). The LOckdown and lifeSTyles in Lombardia (LOST in Lombardia) study was run between 17 and 30 November 2020, on a representative sample of 4,400 older adults (aged 65 and over) from the Lombardy region (Northern Italy).

Participants were randomly selected from a list of 30,000 households, representative of the families in Lombardy in terms of province and size of municipality. A quota approach was employed to assure the representativeness of the older Lombardy population in terms of sex, age, and province of residence. The study protocol was approved by the coordinating group's ethical committee (EC of Fondazione IRCCS Istituto Neurologico Carlo Besta, File number 76, October 2020). All individuals gave their informed consent to participate in the study.

Trained interviewers administered by telephone a questionnaire including information on socio-demographic characteristics, such as age and sex. SARS-CoV-2 infection by was assessed by respondents who self-reported the method of virus identification (i.e., rhino pharyngeal swab, serological test or based on clear symptoms but without a diagnosis).

A specific section of the questionnaire focused on chronic conditions, including tinnitus and hearing loss. Respondents were asked: (i) whether they were currently affected by tinnitus and/or hearing loss, (ii) for those affected, the year of first diagnosis by a physician, and (iii) whether their condition had worsened, improved, or did not change during the COVID-19 emergency, comparing their conditions at the time of the interview (autumn 2020) with the previous year (autumn 2019).

## Statistical Analysis

We employed descriptive statistics and calculated incidence rates (IRs) and their corresponding 95% confidence intervals (CIs), using Fisher's exact method for tinnitus and hearing loss. We used a Chi-square test to compare incidence rates in 2020 vs. the mean incidence rate of the previous two decades (1999–2019). To analyze the relationship between sex and age with tinnitus and hearing loss, we computed odds ratios (ORs) and 95% CIs through unconditional multiple logistic regression models, after adjustment for sex, age and level of education. All analyses considered a statistical weight to ensure that the sample was representative of the general older population of the Lombardy region in terms of sex, age, and province of residence. The software SAS 9.4 (Cary, North Carolina, USA) was used for statistical analyses.

## Results

Out of 4,400 individuals, 358 (8.1%) reported a diagnosis of tinnitus and 463 (10.5%) of hearing loss (Table 1). No statistically significant relationship was found between sex and tinnitus, but tinnitus increased with increasing age (*p* for trend <0.001). Hearing loss was reported less frequently by women than men (OR 0.80; 95% CI: 0.66–0.98) and increased with increasing age (*p* for trend <0.001). Among individuals reporting hearing loss, a percentage of 14.9% people reported tinnitus, while in the group of individuals without hearing loss, only 7.3% reported a perception of tinnitus.

**Table 1:** Distribution of older adults (≥65 years) in Lombardy region (Northern Italy) according to having a diagnosis of tinnitus or hearing loss, by sex and age.

**Characteristics**	***N***	**Tinnitus**		**Hearing loss**	
		**%**	**OR (95% CI)**	**%**	**OR (95% CI)**
Total	4,400	8.1		10.5	
Sex					
Men	1,902	8.9	1.00°	11.2	1.00°
Women	2,498	7.4	0.82 (0.66–1.02)	9.9	**0.80 (0.66-0.98)**
Age group (years)					
65–69	1,289	7.0	1.00°	5.9	1.00°
70–74	838	6.4	0.93 (0.66–1.32)	9.5	**1.63 (1.17–2.27)**
75–79	1,188	8.1	1.23 (0.91–1.67)	11.1	**1.95 (1.45–2.62)**
80–84	739	9.7	**1.59 (1.14–2.21)**	13.9	**2.47 (1.79–3.40)**
85+	346	12.6	**2.23 (1.50–3.30)**	20.3	**3.85 (2.68–5.52)**
P for trend			**<0.001**		**<0.001**

Corresponding odds ratios* (OR) and 95% confidence intervals (CI). LOST in Lombardia, 2020. *Estimated by multiple logistic regression models after adjustment for sex, age and education. Statistically significant estimates at 0.05 level are in bold. °Reference category.

**Figure 1:**
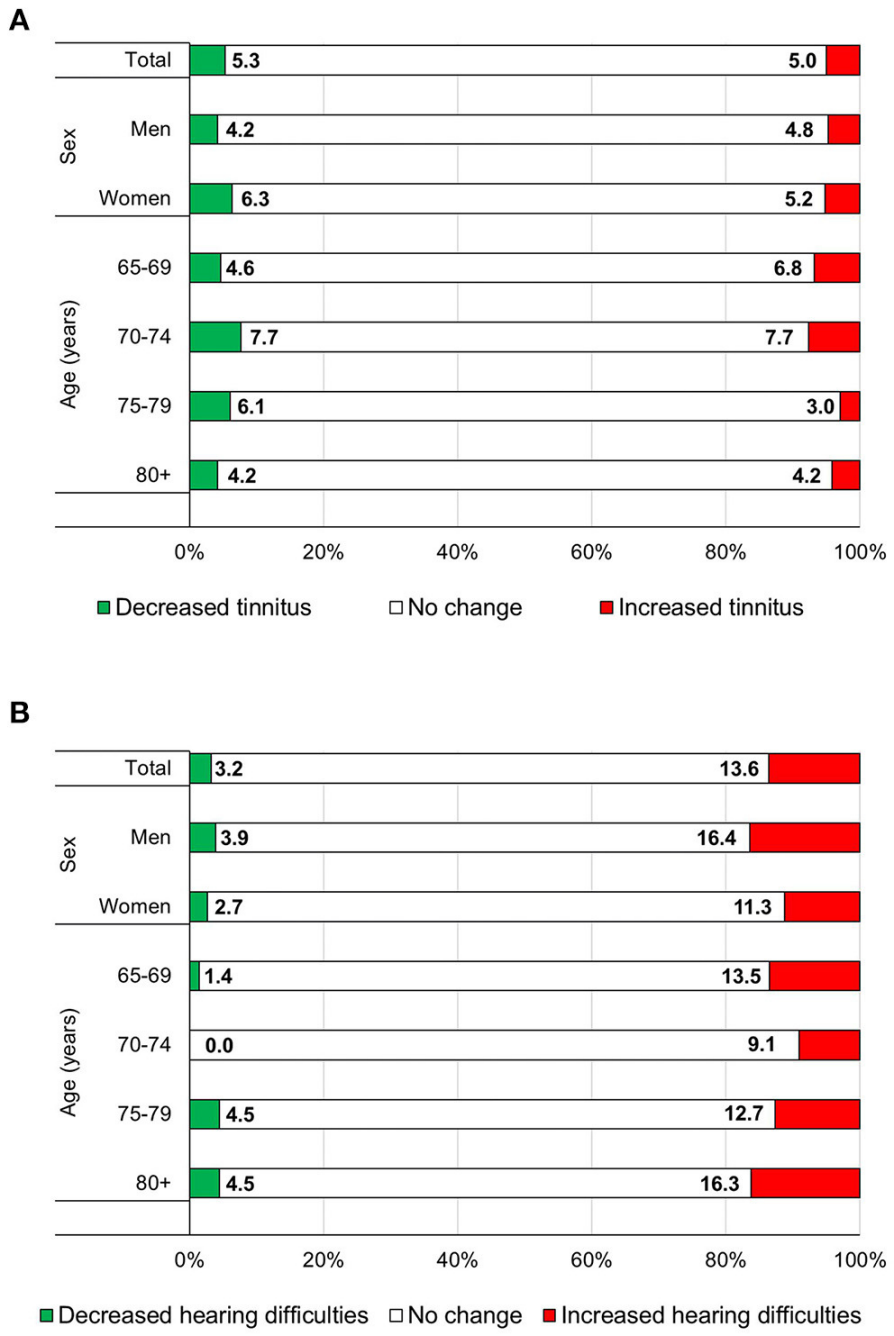
Distribution of individuals aged 65 years or more from the Lombardy region (Northern Italy) with a diagnosis of tinnitus (*n* =3 58) **(A)** or hearing loss (*n* = 463) **(B)**, according to the changes in their condition (decreased or increased) during the COVID-19 pandemic (autumn 2020 compared to autumn 2019), overall and by sex and age group. LOST in Lombardia, 2020.

Among individuals with a diagnosis of tinnitus (8% of the whole sample), 5.3% reported a decrease in symptom severity while 5.0% reported tinnitus worsened in 2020 compared to 2019 (Figure 1). The proportion of tinnitus patients with worsening of the symptom was similar with that of those with improvement. During the COVID-19 pandemic, among individuals reporting a diagnosis of hearing loss 3.2% reported improved hearing while 13.6% noted an increase in hearing problems. More people had worsened hearing loss than those who had improvement.

The IR for tinnitus was 36.0 per 10,000 person-years (95% CI: 32.6–40.0) in 1999–2019 and 14.8 (95% CI: 5.4–32.3) in 2020 (*p* = 0.026). The IR for hearing loss was 50.1 (95% CI: 46.1–54.2) in 1999–2019 and 32.9 (95% CI: 17.5–56.3) in 2020 (*p* = 0.134; data not shown in tables).

Of the 358 individuals with tinnitus, 16 (4.5%) reported a diagnosis of COVID-19. None of them showed any change in its severity (Supplementary Table 1). Of the 463 individuals with hearing loss, 26 (5.6%) reported a diagnosis of COVID-19. Of these, 19 (73.1%) had no change and 7 (26.9%) reported a worsening in the severity of hearing loss (*p* = 0.042 compared to no COVID-19 patients). This association resulted in a crude OR of 2.5 (95% CI 1.0–6.2).

None of the participants with COVID-19 reported a first diagnosis of either tinnitus or hearing loss in 2020 (data not shown in tables).

## Discussion

In this representative sample of older adults from Northern Italy, we found that among individuals reporting a diagnosis of tinnitus (8% of the whole sample), in 5% the symptom improved and in 5% it worsened in autumn 2020 (i.e., during the COVID-19 pandemic) compared to 2019. Among individuals reporting a diagnosis of hearing loss (10% of the whole sample), in 3% their condition improved and in 14% it worsened.

Our findings on the role of the COVID-19 pandemic on tinnitus severity contrast with current evidence suggesting a worsening of tinnitus due to the pandemic (11). In a cohort of 3,103 tinnitus patients, 32% worsened and only 1% improved the severity of their tinnitus (5). In our study, the large majority (90%) of people reporting a tinnitus diagnosis did not experience any change in their perception of its severity and the number of those with worsening was the same as those with improved tinnitus severity.

However, we confirm a possible role of the COVID-19 pandemic on the perception of a worsening of the severity of hearing difficulties (11, 13). We were unable to confirm the hypothesis that women would have more severe exacerbation of tinnitus as a result of the pandemic and its detrimental mental health consequences (4). In fact, our data on hearing loss indicated that the deterioration from the previous year was more evident in men. This is consistent with the worsening of the disorder with age in men more than women (20, 21).

To our knowledge, this is the first study investigating changes in the incidence rates of tinnitus and hearing loss due to the COVID-19 pandemic (11). Although based only on six new cases of tinnitus and thirteen of hearing loss, in 2020 we did not find any increase in its incidence rate, for tinnitus or hearing loss, compared to the previous years. Tinnitus incident cases were in fact significantly lower in 2020 compared to the past. Our results are partially explained by the fact that our survey was conducted in November, thus the year 2020 counted for only eleven months. Moreover, the exceptionality of the pandemic might have served as a barrier for new diagnoses of tinnitus and hearing loss in 2020. In fact, during the COVID-19 pandemic diagnoses of common conditions decreased substantially (22), in Italy specifically regarding cancers (23–25), retinal disorders (26), and cardiovascular diseases (27). In Italy alone this resulted in 12.5 million missing diagnostic tests, 20.4 million blood tests, 13.9 million specialist consultations, and over a million hospital admissions (28).

Among tinnitus patients infected by SARS-CoV-2, none reported changes in their tinnitus status, suggesting that the infection has limited impact, if any, on tinnitus severity. For hearing loss, the proportion of individuals infected by SARS-CoV-2 reporting a worsening of their audiological impairment was higher than those with no infection, although the large majority of SARS-CoV-2 positive individuals reported no change in hearing loss severity. Moreover, none of the COVID-19 patients indicated a concurrent diagnosis of either tinnitus or hearing loss in 2020, thus suggesting that the SARS-CoV-2 infection had no substantial impact on the severity of either tinnitus or hearing loss.

We acknowledge several limitations in our study, including those inherent to its cross-sectional design. We were therefore unable to demonstrate any causal relationship. Moreover, the sample size, although large enough to represent the geriatric population of the Lombardy region, was inadequate to derive robust estimates in selected subpopulations. For example, the incidence rates of tinnitus and hearing loss in 2020 were based on only 6 and 13 cases, respectively. As a telephone-based survey, we introduced an indirect selection bias because only telephone owners were included in our population. However, this was the most accurate mode of data collection in the pandemic period, where contacts had to be kept to a minimum, particularly for the elderly who are less likely to participate in online surveys.

Another limitation is that both tinnitus and hearing loss diagnoses were self-reported, and validated questionnaires were not administered to assess tinnitus or hearing loss. However, to our knowledge, this is the first representative study specifically undertaken on the geriatric population during the COVID-19 pandemic.

If generalized to the whole population of Lombardy, our estimates amount to more than 185 and 240 thousand older people, respectively, being diagnosed with tinnitus and hearing loss. Our findings do not appear to support the hypothesis that the COVID-19 pandemic as a societal stressor has enhanced the severity or incidence of tinnitus. The worsening of hearing difficulties between 2019 and 2020 may totally or partially be explained by a physiologic deterioration of the condition in the 1-year span, and by the fact that the patients had to cope with their illness at a time when face-masks prevented them from reading lips, and generally made it harder to hear each other's words.

## Data Availability Statement

The raw data supporting the conclusions of this article will be made available by the authors, without undue reservation.

## Ethics Statement

The studies involving human participants were reviewed and approved by EC of Fondazione IRCCS Istituto Neurologico Carlo Besta. The patients/participants provided their written informed consent to participate in this study.

## Lost in Lombardia Project Investigators

Silvano Gallus, Cristina Bosetti, Carlotta Micaela Jarach, Alessandra Lugo, Chiara Stival: Istituto di Ricerche Farmacologiche Mario Negri IRCCS, Milan, Italy
Gianluca Serafini, Andrea Amerio, Mario Amore: Università di Genova, Genoa, Italy
David Stuckler, Roberto De Sena, Simone Ghislandi, Yuxi Wang: Università Bocconi, Milan, Italy
Licia Iacoviello^*^, Marialaura Bonaccio^*^, Francesco Gianfagna°: Università degli Studi dell'Insubria, Varese, Italy
^*^IRCCS Neuromed, Pozzilli, Italy
°Mediterranea Cardiocentro, Napoli, Italy
Anna Odone, Carlo Signorelli, Giansanto Mosconi, Giacomo Vigezzi: Università di Pavia, Pavia, Italy and Università Vita-Salute San Raffaele, Milan, Italy
Luca Cavalieri d'Oro, Magda Rognoni, Luca Paroni: Agenzia per la Tutela della Salute della Brianza, Monza, Italy
Alberto Zucchi, Roberta Ciampichini: Agenzia per la Tutela della Salute di Bergamo, Bergamo, Italy

## Author Contributions

CMJ: conceptualization, methodology, formal analysis, writing–original draft, and visualization. AL: supervision and writing-review and editing. CB, AA, LC, LI, AO, DS, and AZ: writing-review and editing and funding acquisition. CS, PB, WG, CC, and WS: writing-review and editing. SG: conceptualization, methodology, supervision, writing-review and editing, and funding acquisition. LOST in Lombardia Study Investigators: funding acquisition. All authors contributed to the article and approved the submitted version.

## Funding

The project is funded by a research grant of the DG-Welfare of Lombardy Region (Call: Progetti di ricerca in ambito sanitario connessi all'emergenza COVID-19; DGR n. XI/3017) and by a grant of the AXA (AXA Research Fund–Call for Proposals COVID-19). The work of AL and SG is partially supported by Unification of Treatments and Interventions for Tinnitus Patients–UNITI project, which has received funding from the European Union's Horizon 2020 Research and Innovation Programme, Grant Agreement Number 848261; the work of CMJ and SG is partially supported by Tinnitus Genetic and Environmental Risks–Tiger project, which has received funding from the European Union's Horizon 2020 Research and Innovation Programme, Grant Agreement Number GNP-182.

## Conflict of Interest

The authors declare that the research was conducted in the absence of any commercial or financial relationships that could be construed as a potential conflict of interest.

## Publisher's Note

All claims expressed in this article are solely those of the authors and do not necessarily represent those of their affiliated organizations, or those of the publisher, the editors and the reviewers. Any product that may be evaluated in this article, or claim that may be made by its manufacturer, is not guaranteed or endorsed by the publisher.
