# Supplementary material for: The Impact of COVID-19 Confinement on Tinnitus and Hearing Loss in Older Adults: Data From the LOST in Lombardia Study
Source: Front Neurol. 2022 Mar 7;13:838291. doi: 10.3389/fneur.2022.838291 (PMC8940241; doi:10.3389/fneur.2022.838291)
Supplement: Supplementary file 1 [file Table_1.DOCX]

Supplementary Material

**Supplementary Table 1:** Supplementary Table 1: Distribution of 358 individuals with a diagnosis of tinnitus and 463 individuals with a diagnosis of hearing loss, from the population aged ≥65 years from Lombardy region (northern Italy), according to changes in their condition during COVID-19 pandemic (autumn 2020 compared to autumn 2019), by sex, age and COVID-19 diagnosis. LOST in Lombardia, 2020.

| Characteristics | Tinnitus | | | | Hearing loss | | | |
| --- | --- | --- | --- | --- | --- | --- | --- | --- |
|  | N | Improved | Not changed | Worsened | N | Improved | Not changed | Worsened |
| Total | 358 | 19 (5.3) | 321 (89.7) | 18 (5.0) | 463 | 15 (3.2) | 385 (83.2) | 63 (13.6) |
|  |  |  |  |  |  |  |  |  |
| Sex |  |  |  |  |  |  |  |  |
| Men | 166 | 7 (4.2) | 151 (91.0) | 8 (4.8) | 207 | 8 (3.9) | 165 (79.7) | 34 (16.4) |
| Women | 192 | 12 (6.3) | 170 (88.5) | 10 (5.2) | 256 | 7 (2.7) | 220 (85.9) | 29 (11.3) |
|  |  |  |  |  |  |  |  |  |
| Age group (years) |  |  |  |  |  |  |  |  |
| 65-69 | 88 | 4 (4.6) | 78 (88.6) | 6 (6.8) | 74 | 1 (1.4) | 63 (85.1) | 10 (13.5) |
| 70-74 | 52 | 4 (7.7) | 44 (84.6) | 4 (7.7) | 77 | 0 (0.0) | 70 (90.9) | 7 (9.1) |
| 75-79 | 99 | 6 (6.1) | 90 (90.9) | 3 (3.0) | 134 | 6 (4.5) | 111 (82.8) | 17 (12.7) |
| 80-84 | 74 | 4 (5.4) | 67 (90.5) | 3 (4.1) | 105 | 6 (5.7) | 83 (79.1) | 16 (15.2) |
| ≥85 | 45 | 1 (2.2) | 42 (93.3) | 2 (4.4) | 73 | 2 (2.7) | 58 (79.5) | 13 (17.8) |
|  |  |  |  |  |  |  |  |  |
| COVID-19 diagnosis |  |  |  |  |  |  |  |  |
| No | 342 | 19 (5.6) | 305 (89.2) | 18 (5.3) | 437 | 15 (3.4) | 366 (83.8) | 56 (12.8) |
| Yes | 16 | 0 (0.0) | 16 (100.0) | 0 (0.0) | 26 | 0 (0.0) | 19 (73.1) | 7 (26.9) |
